# Advances in Enzyme Induced Carbonate Precipitation and Application to Soil Improvement: A Review

**DOI:** 10.3390/ma15030950

**Published:** 2022-01-26

**Authors:** Ahsan Saif, Alessia Cuccurullo, Domenico Gallipoli, Céline Perlot, Agostino Walter Bruno

**Affiliations:** 1 Université de Pau et des Pays de l’Adour, E2S UPPA, SIAME, 64600 Anglet, France; alessia.cuccurullo@univ-pau.fr (A.C.); celine.perlot@univ-pau.fr (C.P.); 2 Dipartimento di Ingegneria Civile, Chimica e Ambientale, Università degli Studi di Genova, 16145 Genoa, Italy; domenico.gallipoli@unige.it (D.G.); agostinowalter.bruno@unige.it (A.W.B.); 3 Institut Universitaire de France (IUF), CEDEX 05, 75231 Paris, France

**Keywords:** Earthen construction materials, urease enzyme, *Sporosarcina pasteurii*, Enzymatic stabilization, EICP, MICP, EICP reaction rate, sustainable geotechnics, soil improvement, soil wind erosion

## Abstract

Climate change and global warming have prompted a notable shift towards sustainable geotechnics and construction materials within the geotechnical engineer’s community. Earthen construction materials, in particular, are considered sustainable due to their inherent characteristics of having low embodied and operational energies, fire resistance, and ease of recyclability. Despite these attributes, they have not been part of the mainstream construction due to their susceptibility to water-induced deterioration. Conventional soil improvement techniques are generally expensive, energy-intensive, and environmentally harmful. Recently, biostabilization has emerged as a sustainable alternative that can overcome some of the limitations of existing soil improvement methods. Enzyme-induced carbonate precipitation (EICP) is a particularly promising technique due to its ease of application and compatibility with different soil types. EICP exploits the urease enzyme as a catalyst to promote the hydrolysis of urea inside the pore water, which, in the presence of calcium ions, results in the precipitation of calcium carbonate. The purpose of this paper is to provide a state-of-the-art review of EICP stabilization, highlighting the potential application of this technique to field problems and identifying current research gaps. The paper discusses recent progress, focusing on the most important factors that govern the efficiency of the chemical reactions and the precipitation of a spatially homogenous carbonate phase. The paper also discusses other aspects of EICP stabilization, including the degree of ground improvement, the prediction of the pore structure of the treated soil by numerical simulations, and the remediation of potentially toxic EICP by-products.

## 1. Introduction

Growing concerns about global warming and the consequent impact on ecosystems have propelled the search for novel construction methods that can reconcile industrial demands with the preservation of the environment. The promotion of sustainable building practices ranks high on the agenda of policymakers worldwide, as the construction industry alone accounts for 23% of the global carbon dioxide (CO_2_) emissions [1] and Portland cement production alone makes up for almost 6% of these emissions [2,3,4]. New environmentally friendly technologies must therefore be embraced in all areas of civil engineering, including geotechnical engineering.

The use of raw earth as a building material (usually in the form of earthen blocks) has attracted significant research interest due to its abundance, markedly smaller carbon footprint, and impressive thermal properties. The expression “earth” indicates a construction material consisting of a mix of soil and water (uncompacted or compacted), which is used with the least possible transformation. In addition to these advantages, raw earth is a natural, environmentally nonintrusive material that can be locally sourced and easily transported to the construction site. Earthen materials are also recyclable, abundantly available, and, if properly manufactured, offers high strength, excellent hygrothermal properties, and low embodied energy at very low costs. Considering its inherently sustainable characteristics, earthen materials can dramatically reduce the exploitation of natural resources not only during construction but also throughout the service life of buildings by cutting down heating/air-conditioning costs and limiting demotion waste.

Despite its eco-friendly attributes, the earthen construction in engineering practice remains limited mainly because of its sensitivity to moisture ingress and susceptibility to water erosion. Similarly, the industry is wary of adopting sustainable ground improvement practices in the absence of in-depth knowledge regarding these methods. Most of the conventional stabilization methods are, however, expensive, invasive, and ecologically aggressive as they require heavy machinery and make use of soil additives with potentially adverse impacts on groundwater and the surrounding environment in general [5,6,7,8]. For example, the use of acrylamide stabilizers was reported in multiple cases of groundwater poisoning in Japan, where all chemical grouts were banned in 1974 except for sodium silicate ones [5]. It has been reported that when 8–12% of cement is added to the earth mix, the average compressive strength achieved is about 3.5 MPa which is equivalent to the strength of high performance fired brick [9]. Likewise, the addition of about 6% lime to finer soils is shown to increase the unconfined compressive strength to about 2 MPa [10].

Though the addition of cement and lime, based on the current recommendations, has ensured the development of highly durable earthen material and easily implementable ground improvement methods, it has inevitably increased the overall carbon emission and energy consumption associated with the material. Lime treatment is subject to leaching, which can decrease strength gains in soil up to 76% over time [10]. Recent studies related to cement stabilization have also shown that the current cement-stabilized earthen materials have net carbon emissions equivalent to that of a lean concrete and fired brick, reducing the original green credentials of natural soils. Furthermore, the addition of cement and lime poses additional performance issues, such as the material’s reduced hygroscopic and recycling capabilities [11]. Reduced hygroscopic capacity leads to higher operational energies to maintain a favourable indoor environment within the earthen building, while reduced recycling potential means the material is down-cycled and dumped into a landfill at the end of its lifecycle. It is, therefore, necessary for both the researchers and practitioners to seek alternative stabilization techniques that provide required engineering performance without compromising on both hygroscopic and recycling properties of the soils.

In this context, biostabilization methods, including those exploiting ureolytic carbonate precipitation, biofilm formation, biogas generation, and biopolymers, have recently emerged as novel ways of improving ground properties without negatively affecting the environment [12]. Ureolytic-induced carbonate precipitation (UICP) has demonstrated great potential for field applications as it can significantly improve the mechanical properties of weak soils while minimizing ecological impact. UICP exploits the hydrolysis of urea (ureolysis), catalyzed by the urease enzyme, to generate the precipitation of calcium carbonate, which bonds soil particles together, thus increasing strength and reducing void sizes. Ureolytic precipitation of calcium carbonate can be achieved by either exploiting the action of soil microbes that produce the urease enzyme (microbially induced carbonate precipitation, i.e., MICP) or adding the urease enzyme directly to the ground (enzyme-induced carbonate precipitation, i.e., EICP).

Considerable research has been undertaken to develop MICP as a viable ground improvement method, including laboratory studies and large-scale experiments, which have highlighted the advantages but also the drawbacks of this technique. MICP is nowadays a relatively well-established soil improvement method and is supported by sound scientific evidence as summarized by several review articles [13,14,15,16,17,18]. In contrast, EICP has only recently gained traction; thus, there is much room for research and improvement. Recently, Putra et al. [19] and Almajed et al. [20] published interesting review articles related to EICP. However, these studies focused more on the outcome of EICP stabilization rather than the main factors that affect its efficiency. The aim of this paper, therefore, is to review the key developments of EICP stabilization and identify the controlling factors that must be considered in large-scale soil improvement and earthen construction projects. Based on the available literature, this paper identifies EICP as an efficient and viable alternative to other soil stabilization methods, including MICP.

## 2. Background

### 2.1. Ureolytic-Induced Carbonate Precipitation (UICP)

**Figure 1:**
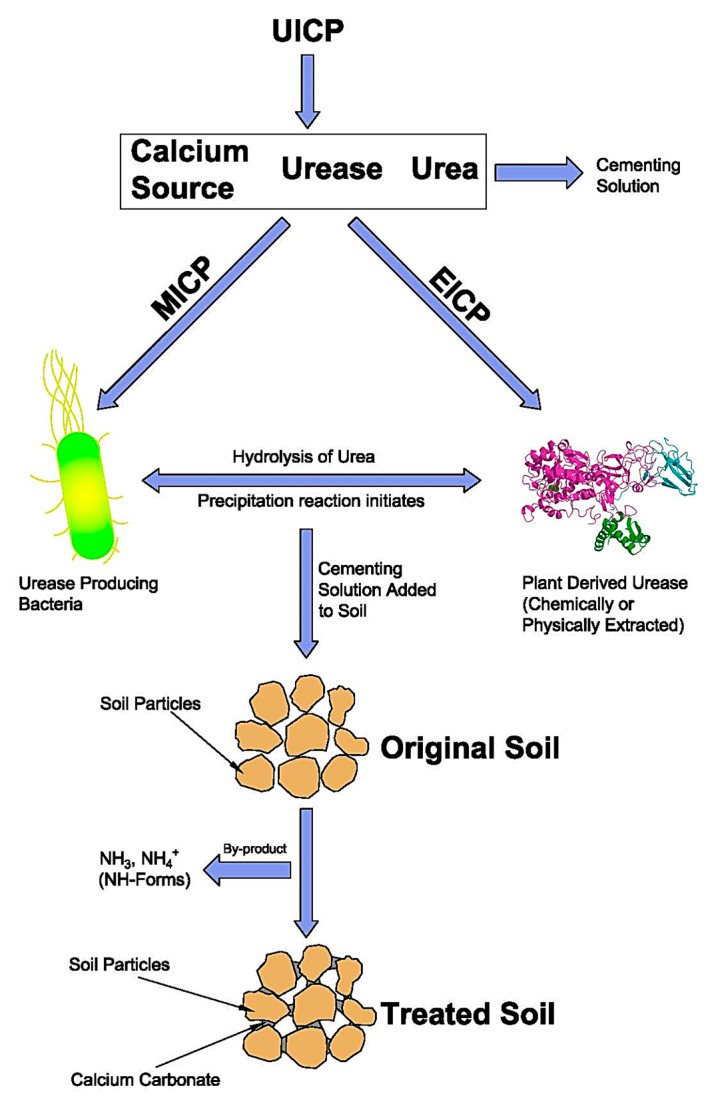
Schematic representation of UICP.

Ground stabilization by ureolytic calcification relies on the dissociation of urea (CH_4_N_2_O) into ammonium (NH_4_^+^) and carbonate ions (CO_3_^2−^) by the urease enzyme, which acts as a reaction catalyst. Carbonate ions from ureolysis react with calcium ions (Ca^+2^) to precipitate calcium carbonate (CaCO_3_) inside the ground. Hydrolysis of urea occurs spontaneously in water at a very slow rate, but the reaction kinetics is sped up by a factor of 10^14^ when the urease enzyme is used as a catalyst [21]. The catalysis of ureolytic reactions can be achieved by exploiting the metabolic activity of urease-producing microbes (as in MICP) or by adding the urease enzyme directly to the reaction medium (as in EICP). These two methods follow similar steps (Figure 1), though the requirements for the successful application of each one can differ to a great extent, as discussed later on.

Equations (1)–(6) summarize the main chemical reactions during UICP stabilization. Equation (1) shows the hydrolysis of urea, which produces ammonia and carbamic acid, thus increasing pH. Equation (2) shows that the carbamic acid is further hydrolyzed into ammonia plus carbonic acid. According to Equations (3) and (4), the reactions then proceed to generate ammonium and carbonate ions alongside hydroxide ions, which further increases the pH of the reaction medium. Equation (5) summarizes the above four reactions and indicates that the hydrolysis of one mole of urea produces two moles of ammonium and one mole of carbonate ions. Finally, Equation (6) indicates that the reaction between carbonate ions and calcium ions leads to the precipitation of calcium carbonate once supersaturation is attained.

(1) $$\mathrm{CO}{\left({\mathrm{NH}}_{2}\right)}_{2}+H_{2}O\to {\mathrm{NH}}_{2}\mathrm{COOH}+{\mathrm{NH}}_{3}$$

(2) $${\mathrm{NH}}_{2}\mathrm{COOH}+H_{2}O\to {\mathrm{NH}}_{3}+H_{2}{\mathrm{CO}}_{3}$$

(3) $$2{\mathrm{NH}}_{3}+2H_{2}O\to 2{\mathrm{NH}}_{4}^{+}+2{\mathrm{OH}}^{-}$$

(4) $$2{\mathrm{OH}}^{-}+H_{2}{\mathrm{CO}}_{3}\to {\mathrm{CO}}_{3}^{-2}+2H_{2}O$$

(5) $$\mathrm{CO}({\mathrm{NH}}_{2}{)}_{2}+2H_{2}O\to 2{\mathrm{NH}}_{4}^{+}+{\mathrm{CO}}_{3}^{-2}$$

(6) $${\mathrm{Ca}}^{+2}+{\mathrm{CO}}_{3}^{-2}\to {\mathrm{CaCO}}_{3}$$

Calcium carbonate precipitates at the contacts between soil grains and in the pore space, thus binding the particles together and filling the intergranular voids. This generally improves the soil properties, including increasing strength and stiffness and decreasing permeability [22]. UICP has also been applied in the preservation of limestone monuments [23,24], the selective filling of voids inside oil reservoirs [25], the removal of contaminants from soils and groundwater systems [26,27,28,29,30,31], the repair of cracked concrete [32,33,34,35], the reduction of ground swelling [36], and the mitigation of soil liquefaction [37,38,39]. Another advantage of UICP stabilization is that the carbonate precipitate is generally durable and dissolves very slowly, i.e., on a geological time scale [40].

The efficiency of ureolytic reactions depends on several factors, including the mode of application of the urease enzyme (i.e., either directly or microbially mediated), the concentration of urea, the source as well as the concentration of calcium ions and urease, the activity of urease, the temperature of the cementing solution (containing urease, urea and calcium source), the pH of the cementing solution, the pH of the soil, the coarseness and mineralogy of the soil, the curing time, and, finally, the chemical composition of the pore water. Additional factors come into play for MICP stabilization because, in this case, the reactions are mediated by the microbes that produce the urease enzyme. It is therefore vital to consider the microbial cultivation time, the amount of microbial solution, the amount of nutrient broth, and the availability of oxygen at the treatment location.

The dependency of UICP stabilization on multiple factors may also constitute an advantage over cement and lime treatment because the reaction rate can be controlled by different process variables such as the pH, the temperature, or the concentration of reactants instead of adding external admixtures. Understanding the role of the different parameters affecting the UICP reaction rate is, therefore, crucial to achieving an effective stabilization of the ground at a large scale.

### 2.2. Microbially Induced Carbonate Precipitation (MICP)

MICP exploits the metabolic activity of ureolytic bacteria to produce the urease enzyme and, hence, to catalyze the precipitation of calcium carbonate according to the previous reactions (Equations (1)–(6)). The most commonly used bacterium for this purpose is *S. pasteurii*, which may be indigenous (i.e., naturally present) or exogenous (i.e., externally injected) to the ground. *S. pasteurii* is an aerobic, alkalophilic, Gram-positive bacterium with relatively high activity and high production of urease enzyme [16,41]. *S. pasteurii* is not pathogenic or genetically modified, does not enclose exchangeable elements that can harm the soil, and is not repressed by ammonium, which is a by-product of the hydrolysis of urea [42,43,44,45]. Ureolytic bacteria use the urease enzyme to hydrolyze urea into ammonia and carbon dioxide, which then diffuse through the microbial cell wall into the surrounding solution [14]. The subsequent reactions leading to the precipitation of calcium carbonate are those already presented in Equations (1)–(6). MICP stabilization has been applied to sandy or cohesionless soils [13,14,46,47,48,49,50] while only a limited number of studies have focused on fine-grained and organic soils [51,52,53]. These investigations have shown that the treatment of soils by MICP produce relatively large gains of strength and a marked reduction of permeability both at a small laboratory scale [54,55,56] and at large scale [22,50,55,57,58,59]. The largest gains of unconfined compressive strength were reported by Whiffin [60] with values as high as 34 MPa in treated soils, while the largest reductions in permeability, between 22% and 75%, were reported by Whiffin et al. [55].

MICP stabilization has also shown few technological bottlenecks, especially when applied to ground stabilization at a large scale. The most common difficulties include the occurrence of bioplugging, the management of on-site bioreactors and the generation of toxic by-products such as ammonium salts [22,61]. Bioplugging consists of the premature calcification of the cementing solution around the injection point, which not only impedes a spatially homogeneous distribution of reactants but also makes further flushing difficult and energy intensive [62]. Much research has therefore focused on developing suitable injection techniques that can increase the spatial homogeneity of the treatment at a large scale. Whiffin et al. [55] performed MICP experiments on sand columns with a diameter of 6.6 cm and height of 5 m concluding that a careful balance must be struck between the solution injection rate and the conversion rate of the reactants to achieve a uniform ground improvement. In an additional study, Van Paassen et al. [58] performed MICP experiments on two types of sand cubes, one having a volume of the order of 1 m^3^ (0.9 × 1.1 × 1 m^3^) and another having a volume of the order of 100 m^3^ (8 × 5.6 × 2.5 m^3^). They measured largely variable levels of unconfined compressive strength, from 0 to 12 Mpa, at different locations inside the treated cubes. Interestingly, however, no accumulation of calcium carbonate was found near the injection point, with a consequent absence of bioplugging, which was attributed to the relatively high injection rate compared to the reaction rate.

Harkes et al. [22] experimented with a stepwise injection method inside sand columns of 6.6 cm diameter and 18 cm height, in which the bacterial solution was injected first, followed by the fixation fluid (calcium salt solution) and, finally, by the fluid containing urea. Although no clear improvement was observed, the authors speculated that the spatial homogeneity of carbonate precipitation could be improved by varying the amounts of bacterial solution and fixation fluid. Ebigbo et al. [59], flushed sand columns, having a diameter of 2.5 cm and a height of 61 cm, with calcium- and urea-free medium between subsequent injection cycles to lower saturation levels and avoid premature clogging. This procedure prevented bioplugging and helped achieve a more homogenous, albeit quantitatively smaller, precipitation of calcium carbonate.

MICP stabilization can also be costly and lengthy as exogenous bacterial colonies must often be cultivated inside a nutrient broth under specific environmental conditions before being injected into the ground. The injection of bacteria into the ground may also interfere with existing microorganisms and alter the ecological balance of the soil, thus producing a long-term negative impact on the surrounding ecosystem [63,64]. Moreover, the subsistence of bacterial colonies at great depths is often hindered by the insufficient supply of oxygen and nutrients, which is highly detrimental to their activity and, hence, carbonate precipitation [14].

Bacteria are often too large to fit inside pores smaller than approximately 0.4 μm, which precludes the application of MICP to fine-grained and highly dense cohesionless soils [65]. This problem may be overcome by exploiting indigenous bacterial colonies, which are naturally present inside the soil, instead of injecting exogenous strains [66,67,68,69]. Even in this case, however, the limitations associated with the chemical composition of the pore water, the absence of oxygen at great depths, and the interference from indigenous microorganisms persist. Rebata-Landa [70] reported that MICP stabilization could be equally ineffective in soils of very low density because the thin calcite layer produced by the ureolytic reactions cannot bridge distant particles, though this limitation would similarly apply to other UICP stabilization methods.

### 2.3. Enzymatic-Induced Carbonate Precipitation (EICP)

EICP has been developed as a sustainable and effective ground stabilization method that can overcome some of the limitations of MICP. In the case of EICP, the urease enzyme is directly introduced into the ground as a reactant alongside urea and calcium ions instead of being produced by the metabolic activity of bacteria, as in MICP. The direct introduction of urease into the soil bypasses the complexities associated with the cultivation and fixation of bacteria, thus circumventing the need for a bioreactor on site. It also sidesteps the difficulties posed by the penury of oxygen at large depths and the interference of indigenous microorganisms inside the soil.

The urease enzyme is a widely occurring hexameric protein found in bacteria, higher order plants, and some vertebrates [62]. The urease molecule has a size of 12 nm [71] and is, therefore, much smaller than bacteria, all of which are bigger than 300 nm, with most of them in the range of 500–5000 nm [62]. For example, the average size of *S. pasteurii* cells is about 2800 nm [72], which is more than two hundred times larger than the size of the urease molecule. Unlike MICP, whose application is restricted to coarse materials, EICP can be employed to stabilize fine-grained and heavily compacted soils, thanks to the relatively small size of the urease molecule, which can fit inside small pores. The enzyme also has a limited lifespan and naturally decomposes inside the soil without disrupting the local ecosystem [73,74]. The urease activity is usually measured in units/gram, where one unit is defined as the amount of enzyme required to hydrolyze 1 μmol urea per minute at a pH of 7.0 and a temperature of 25 °C. Low temperatures negatively affect both the growth of bacterial colonies and the activity of the urease enzyme, which makes EICP more effective than MICP in cold climates as it suffers only from the latter effect but not the former one [75]. These advantageous characteristics explain the recent interest in EICP stabilization as an effective and sustainable method of soil improvement.

Purified urease can be chemically isolated from plants via synthetic processes [76] that are routinely undertaken by chemical manufacturers. Some varieties of beans (e.g., jack beans and soybeans), watermelon seeds, and the pine family are relatively rich in urease enzyme [77]. However, purified urease is expensive at an approximate cost of about EUR 20 per gram (indicative price quoted by commercial providers in 2021) and can prove uneconomical for large scale applications. Because of this, some researchers have advocated the use of crude plant extracts as an economical alternative to purified urease. This includes crude extracts from jack beans [78,79], watermelon seeds [80] and soybeans [81,82].

The following sections discuss recent advances in EICP stabilization research, detailed in the publications listed in Table 1.

## 3. Parameters Affecting EICP Reaction Rate

Similar to MICP, achieving a spatially homogeneous ground treatment via EICP is a challenging task as the formation of fresh precipitate restricts the subsequent transport of the cementing solution to longer distances. The reaction rate plays a pivotal role in governing the spatial distribution of the calcium carbonate and, therefore, the effectiveness of EICP as a ground improvement technique. It has been reported that the rate of precipitation must generally be low so that the reactants take longer to convert into calcium carbonate, allowing the cementing solution to travel farther through the soil pores [22]. The following subsections discuss the effect of the different factors influencing the rate of calcium carbonate precipitation. In principle, the sensitivity of the reaction rate to each one of these factors can be exploited to increase the spatial homogeneity of ground treatment.

### 3.1. Effect of Reactants Concentrations

**Figure 2:**
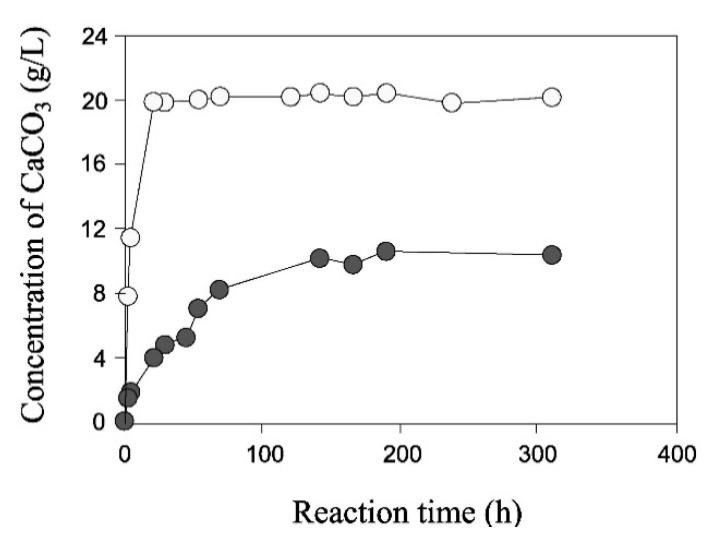
Effect of urease concentration: black curve = urease amount of 0.01 g/L, white curve = urease amount of 0.1 g/L (from Nemati and Voordouw [26], Copyright under Elsevier).

**Figure 3:**
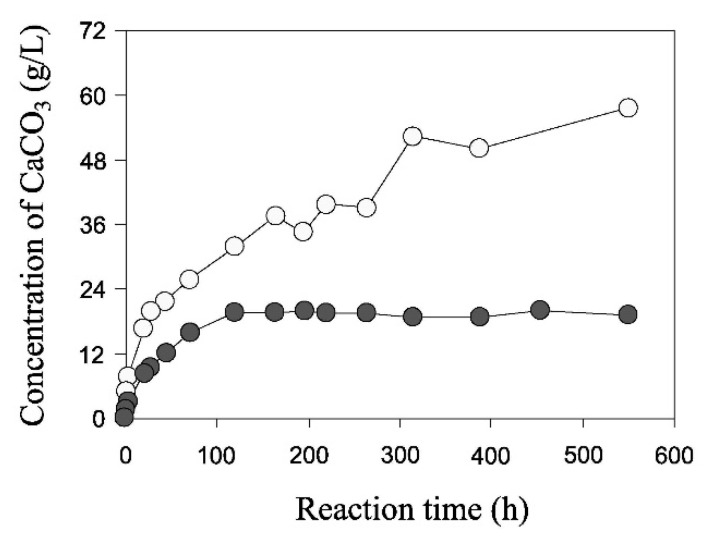
Effect of reactants concentrations: black curve = base solution, white curve = threefold increase of all reactants (Nemati and Voordouw [26], Copyright under Elsevier).

Nemati and Voordouw [26] conducted one of the earliest studies on the application of EICP to the consolidation of sand in oil reservoirs and the detection of contaminants in groundwater systems. They conducted test tube experiments to evaluate the influence of the concentrations of urease, urea, and calcium chloride on the rate and amount of carbonate precipitation, which provided a helpful insight into some of the parameters that affect the efficiency of EICP reactions. For example, they showed that, for fixed concentrations of urea and calcium chloride, an increase in the amount of urease boosts the reaction rate exponentially. Figure 2 shows that a urease amount of 0.1 g/L produces a relatively rapid equalization of the chemical reactions with approximately 20 g/L of carbonate precipitate after only a few hours. In contrast, a smaller urease amount of 0.01 g/L results in a slower reaction rate, achieving equalization after nearly 200 h, with a smaller precipitate quantity of 10 g/L. Figure 3 shows that a proportional increase of all reactants produces a strong augmentation of precipitate accompanied by a marginal growth of the reaction rate. For the base reactant concentration, the precipitation rate is high during the first 50 h and stabilizes after almost 150 h, with a precipitate amount of 16 g/L. A threefold increase of all reactants collectively produces a drastic augmentation of precipitate to almost 60 g/L, though the increase of reaction rate is comparatively smaller with chemical reactions not stabilizing after 600 h.

**Figure 4:**
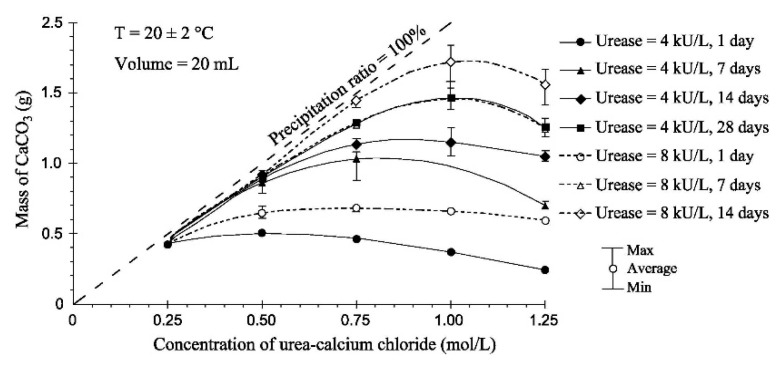
Effect of reactants concentration for different curing times (Carmona et al. [86], Copyright under ICE Publishing).

In an analogous study, Yasuhara et al. [83,84] and Neupane et al. [85] performed test tube experiments with varying amounts of reactants and observed that relatively high concentrations of urea and calcium chloride may restrict the activity of the urease enzyme and may result in reduced carbonate precipitation. This result is also consistent with the work of Carmona et al. [86], who conducted test tube experiments and measured the amount of calcium carbonate precipitate after 1, 7, 14, and 28 days. They concluded that, for a given amount/activity of urease (kilo units/litre, kU/L), there is an optimum concentration of reactants (urea and calcium chloride) at which the maximum carbonate precipitation occurs for a fixed curing time (Figure 4). The decrease of carbonate precipitation beyond this optimum concentration is attributed to the drop in pH produced by higher urea amounts, which lead to a reduction of reaction rate, as detailed later in Section 3.6.

**Figure 5:**
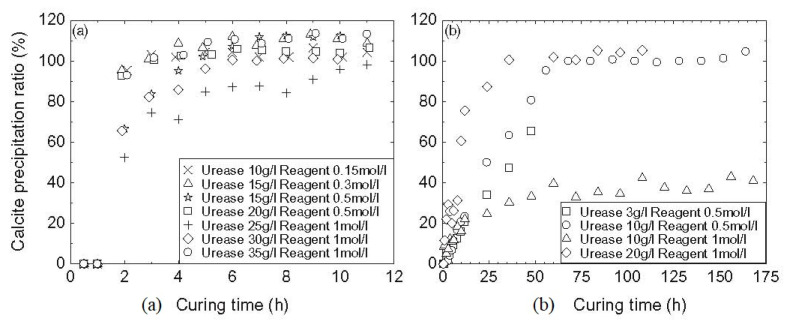
Precipitation ratios with respect to curing time for different concentrations of reactants (**a**) PR greater than 90% for all solutions (**b**) PR obstructed in some solutions (Simatupang and Okamura [87], Copyright under Elsevier).

Simatupang and Okamura [87] argued that the decrease of carbonate precipitation beyond the optimum concentration level may not be permanent and, if a longer curing time is allowed, further conversion of reactants into products would take place. They supported this argument with the experimental data shown in Figure 5a, which indicates that distinct cementing solutions of different concentrations can all reach a precipitation ratio (PR, defined as the ratio between actual and theoretical amounts of precipitate) of at least 90% as curing time progresses. Nonetheless, the authors also acknowledged that there are specific concentrations that cannot achieve the complete conversion of reactants at equilibrium, as shown in Figure 5b for 10 g/L of urease and 1 mol/L of reactants. This incomplete conversion may again be explained by the very high concentration of urea relative to the amount/activity of the urease enzyme, which causes a drop in pH.

Both the activity and concentration of urease play a key role in governing the efficiency of the chemical reactions that lead to carbonate precipitation. Therefore, these two variables should be ideally combined into a single parameter to allow an accurate evaluation of the catalyst effect of the enzyme at different dilution levels and curing times.

### 3.2. Effect of Urease Source

The source and extraction method have a direct impact on the catalyst activity of urease, which in turn influences the amount of enzyme needed to achieve a certain level of carbonate precipitation; the greater the activity, the smaller the amount required. This is important because the price of the urease enzyme accounts for a substantial share, between 57% and 98%, of the total cost of EICP stabilization [88]. El-Hefnawy et al. [89] reported that purified urease, which had been chemically extracted from Pisum Sativum L. seeds (green peas), exhibits a relatively high average activity of 5833 U/mg. Similarly, Krajewska B. [90] showed that purified urease chemically extracted from soybeans, pigeon pea, and jack beans exhibits activity ranges of 650–800 U/mg, 3120 U/mg, and 2700–3500 U/mg, respectively. Dilrukshi et al. [8] compiled an exhaustive list of urease activity ranges for different plant species, which can be used as a reference for future EICP studies.

**Figure 6:**
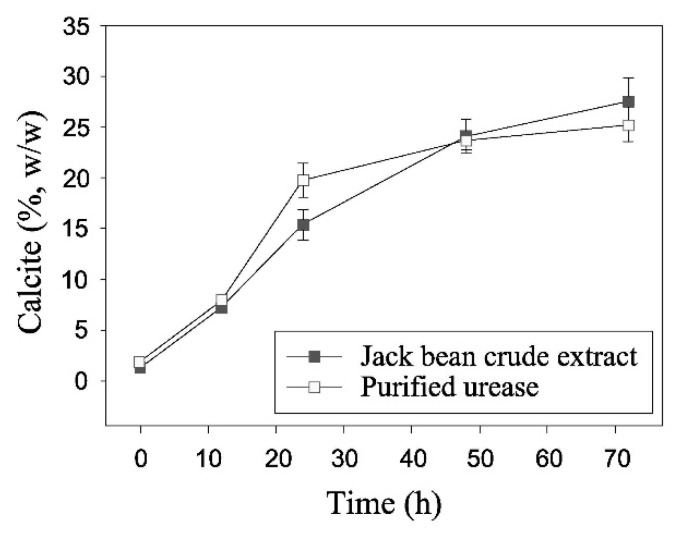
Comparison of calcium carbonate precipitation from crude centrifuged jack beans extract and purified urease (Nam et al. [78], Copyright under Springer Nature).

Recent investigations have also demonstrated the viability of alternative and more economical separation methods based on the centrifugation of urease-rich plant seeds inside a standard juice extractor. Nam et al. [78] showed that purified urease and crude centrifuged jack beans juice have similar enzymatic efficiency and can produce similar amounts of carbonate precipitation over a period of 72 h (Figure 6). The specific activity of the crude urease extract (41.08 U/mg of protein) was found to be only slightly lower than that of purified urease (43.57 U/mg of protein), making the former an equally effective but significantly more economical option compared to the latter.

Dilrukshi et al. [80] compared the activity of the urease from stirred (500 rpm) and centrifuged (10,000 rpm) crude extracts of watermelon seeds soaked in distilled water at 25 °C. The activity of the centrifuged extract was greater than that of the stirred one and increased almost linearly with the growing amount of watermelon seeds per litre of water. Cuccurullo et al. [82] demonstrated that a soybean crude extract may be an effective source of urease enzyme by comparing the durability of treated and untreated soil samples compacted at the standard Proctor optimum moisture content. All samples were subjected to immersion tests resulting in a mass loss of 42% for the untreated material compared to only 13% for the treated one. Baiq et al. [91] compared the amount of carbonate precipitation generated by purified urease chemically isolated from jack beans with that generated by a crude extract of cabbage and soy pulp. It was found that the amount of precipitate produced by the crude extract was about 40–60% of the precipitate produced by the purified urease.

Past studies have generally pointed out that crude plant extracts can provide an accessible and viable source of urease for large-scale geotechnical and structural applications, though the extraction method remains an important factor affecting enzymatic activity.

### 3.3. Effect of Calcium Source

Calcium carbonate commonly exists in three natural forms, namely calcite, aragonite, and vaterite [23], with calcite being the most stable while aragonite and vaterite being metastable under room conditions. The precipitation of calcium carbonate inside a concentrated solution of calcium ions and carbonate ions usually involves three consecutive steps: a) the formation of amorphous calcium carbonate characterized by low stability and high solubility, b) the transformation of amorphous calcium carbonate into vaterite, and c) the transformation of thermodynamically unstable vaterite into stable calcite [92,93,94]. MICP studies have reported the formation of different calcium carbonate crystals, depending on the salt used as a source of calcium ions. Calcium chloride leads to the precipitation of rhombohedral shaped calcite crystals [95,96,97], while lamellar shaped vaterite crystals are produced by calcium acetate. More complex vaterite crystals with a spherical shape are precipitated from calcium lactate and calcium gluconate [98].

**Figure 7:**
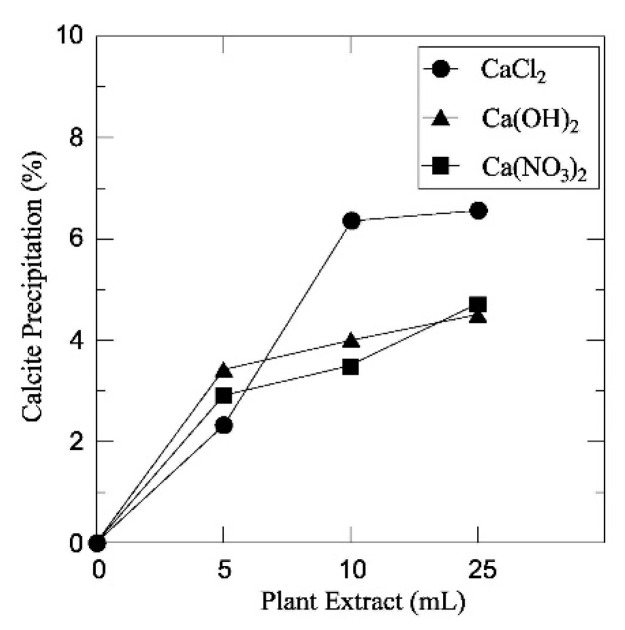
Comparison of the amounts of carbonate precipitation resulting from different calcium sources (Park et al. [79], Copyright under ASCE).

Different calcium salts exhibit different solubility levels in free water, thus influencing the number of calcium ions available for the ureolytic reactions. Previous MICP studies have reported that calcium chloride is the most effective source of calcium ions to maximize carbonate production [97,99]. Similarly, EICP leads to the precipitation of different amounts of calcium carbonate and the formation of different types of crystals depending on the source of calcium ions. Park et al. [79] used a crude centrifuged jack beans extract (plant extract, mL) as a urease source to explore the influence of the type of calcium salt on the efficiency of EICP. Three different calcium salts (i.e., calcium hydroxide Ca(OH)_2_, calcium nitrate Ca(NO_3_)_2_, and calcium chloride CaCl_2_) were separately added in equal amounts to the crude extract along with urea before being mixed with sand to prepare samples for mechanical testing. Unconfined compression tests showed that calcium chloride generated the greatest levels of unconfined compressive strength up to 317 kPa, while calcium hydroxide and calcium nitrate generated a maximum strength of 244 kPa and 253 kPa, respectively. Moreover, calcium chloride produced the highest percentage of carbonate precipitation (amount of precipitate measured with respect to the total sample mass), followed by calcium hydroxide and calcium nitrate, both of which had almost equal percentages of carbonate precipitation (Figure 7). These results are also consistent with the almost 100 times larger solubility at room temperature of calcium chloride compared with calcium hydroxide and calcium nitrate, though the authors provided no information about which type of crystals were generated by the three calcium salts.

### 3.4. Effect of Magnesium Salts

Putra et al. [100] proposed a method to delay EICP reactions rate by partly replacing calcium chloride (CaCl_2_) with magnesium chloride (MgCl_2_) while keeping the total molar concentration of both salts equal to 0.5 mol/L. Different cementing solutions were prepared using varying ratios of calcium chloride and magnesium chloride concentrations along with constant amounts of urea and urease. Test tube experiments were carried out by first mixing the urease enzyme with water and subsequently adding the reactants (i.e., urea, calcium chloride, and magnesium chloride). The partial substitution of calcium chloride with magnesium chloride generated a time lag in the evolution of pH, which was measured at 0, 1, 2, 3, 6, 7, 8, 9, and 10 h after mixing the reactants. For solutions without magnesium chloride, the pH started to decrease after about 1 h and stabilized after 3 h. In comparison, for solutions with magnesium chloride, the pH decreased after 2 to 4 h and stabilized after 8 to 10 h depending on the concentration ratio of the two salts. The results hinted at slower carbonate precipitation with increasing percentages of magnesium chloride due to the simultaneous availability of magnesium and calcium ions for carbonate bonding, which resulted in a delay of the reaction rate. The precipitation ratio increased up to 90% in solutions containing 10–20% of magnesium chloride, though a further increase in magnesium chloride reduced the amount of calcium carbonate production. The increase of magnesium salt also reduced the size of carbonate crystals, decreased calcite content, and promoted the growth of aragonite with a progressive shift from crystalline to an amorphous structure.

**Figure 8:**
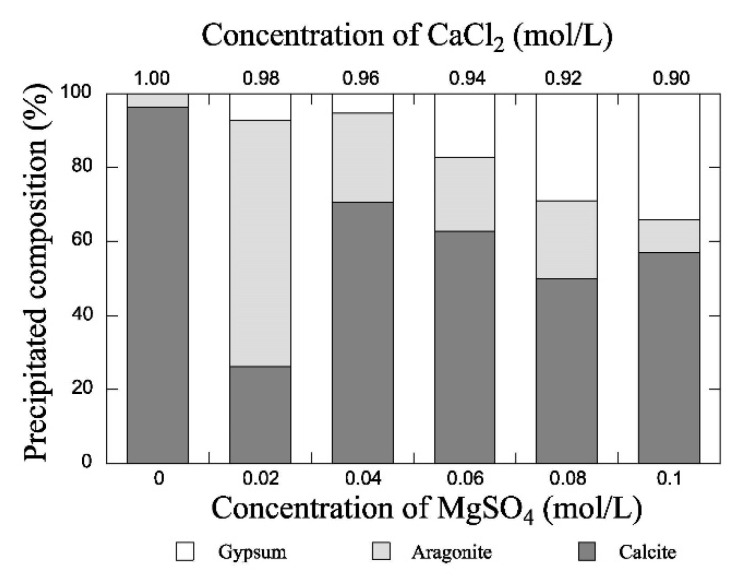
Effect of the concentration of magnesium sulphate on the precipitate composition (Putra et al. [101], Copyright under MDPI).

A similar study by Putra et al. [101] found that growing concentrations of magnesium sulfate (MgSO_4_) increased the precipitation ratio to values larger than 100% while leading to the formation of larger fractions of gypsum and smaller fractions of aragonite (Figure 8). The authors also argued that growing concentrations of magnesium sulfate increased the precipitation rate, which in turn promoted the transition of precipitation products from aragonite to gypsum.

### 3.5. Effect of Temperature

**Figure 9:**
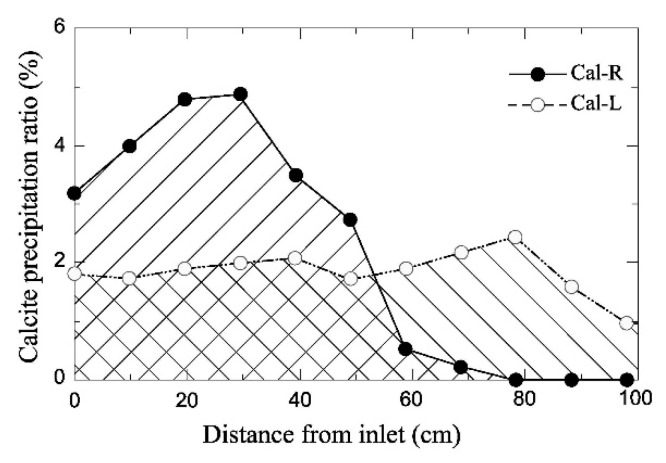
Distribution of calcium carbonate in sand columns permeated by cementing solutions at 5 °C (Cal-L) and 23.5 °C (Cal-R) (Neupane et al. [102], Copyright under Elsevier).

Neupane et al. [102] investigated the effect of temperature on EICP inside sand columns of 1 m height and 5 cm diameter. Cementing solutions at two different temperatures of 5 °C and 23.5 °C were flushed across the columns with a constant flow rate of 80 mL/min from bottom to top. The CO_2_ Volume Evaluation (CVE) method was applied to measure the amount of carbonate precipitation at different distances from the injection point. Figure 9 shows that the cementing solution at 23.5 °C (Cal-R) produced a relatively high amount of calcium carbonate near the injection point, which decreased with growing distance becoming negligible beyond 60 cm. Conversely, the cementing solution at 5 °C (Cal-L) generated a more uniform distribution of calcium carbonate, though the amount of precipitate was less than in the previous case.

**Figure 10:**
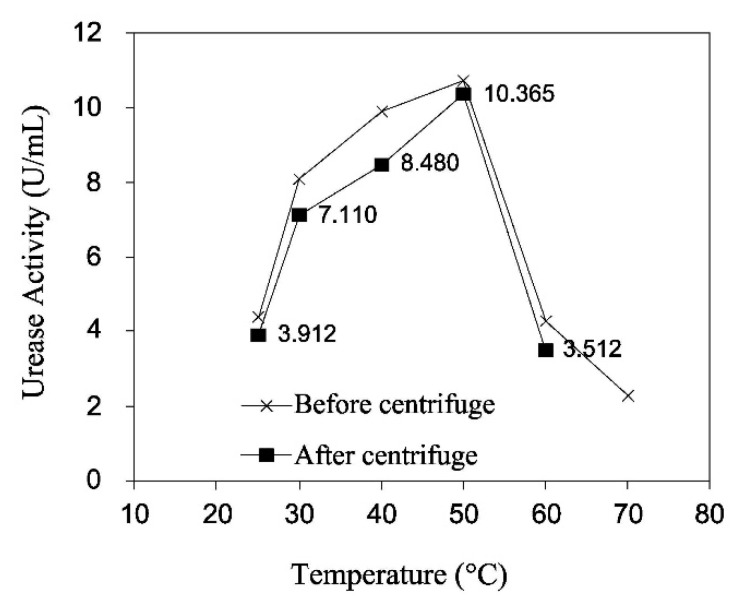
Activity of urease extract from stirred (before centrifuge) and centrifuged (after centrifuge) watermelon seeds at different temperatures (Dilrukshi et al. [80], Copyright under Elsevier).

**Figure 11:**
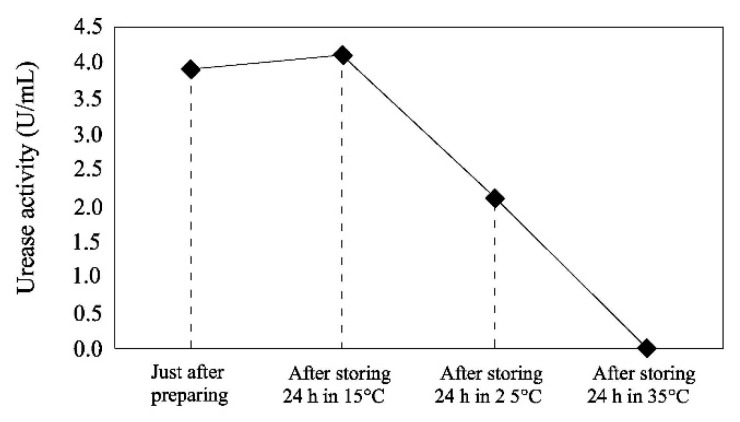
Decay of activity of urease extracts from watermelon seeds during storage for 24 h at different temperatures (Dilrukshi et al. [80], Copyright under Elsevier).

The strong correlation between urease activity and temperature is the main cause of the thermal variations of EICP. Dilrukshi et al. [80] measured the activity of crude urease extracts from watermelon seeds at different temperatures, from 25 °C to 70 °C, using the indophenol method [103]. Their results confirmed the thermal dependency of urease activity, which increased with growing temperature up to 50 °C and was followed by a rapid decrease afterwards. The results were very similar irrespective of whether the urease extract was obtained by stirring or centrifuging the watermelon seeds (Figure 10). The study also showed that the activity of the crude extract decreased with time and that the rate of decay was more pronounced at higher temperatures (Figure 11).

The above results align with those of Nemati and Voordouw [26], who observed full conversion of reactants after 120 h of curing at temperatures from 30 °C to 50 °C but only 70% conversion after 300 h of curing at a temperature of 20 °C. Sun et al. [75] performed acid digestion tests to measure the carbonate content of EICP stabilized soil samples cured for eight days at temperatures between 10 °C and 30 °C observing a smaller amount of precipitation and more marked crystal nucleation at lower temperatures.

The above results also indicate that the spatial uniformity of carbonate precipitation may be increased, especially in large scale projects, by controlling the temperature of the treated area or cementing solution.

### 3.6. Effect of pH

The pH of the reaction medium is one of the crucial abiotic factors influencing the activity of the urease enzyme [104]. Rohy et al. [105] argued that slight acidification of the cementing solution could delay the EICP reaction rate. To support this conclusion, they mixed a crude urease extract from jack beans with different amounts of urea and calcium chloride at a constant molarity ratio of 1:0.67 to generate three cementing solutions, having urea concentrations of 1M, 2M, and 3M, respectively. They observed that the pH of the three cementing solutions decreased with the augmentation of urea resulting in pH values of 5.69, 4.94, and 4.46 for the 1M, 2M, and 3M solutions, respectively. The solutions were then mixed with sandy soil to produce cylindrical samples of 10 cm height and 5 cm diameter, which were cured at room temperature and humidity for three days before being subjected to unconfined compression tests. Test results showed an average strength of 219 kPa for the 1M solution samples, which increased to 314 for the 2M solution samples and 504 kPa for the 3M solution samples. Additional samples prepared with the 3M solution were tested after longer curing times of 7 and 14 days showing substantial increases of strength to 1176 and 3000 kPa, respectively. This significant increase of strength over time is consistent with a slower precipitation rate at lower pH values requiring longer intervals to attain equilibrium.

Cui et al. [106] used hydrochloric acid (HCl) to adjust the pH of different cementing solutions employed for stabilizing soil specimens. The specimens were cured at room temperature (25 ± 1 °C) for 24 h before being subjected to unconfined compression tests to measure strength and acid digestion tests to measure carbonate content. Samples treated with cementing solutions at low pH exhibited a more uniform carbonate distribution and higher levels of unconfined compressive strength. Using similar experimental techniques, Sun et al. [75] showed that the precipitation rate increases as the pH of the cementing solution grows from 7.0 to 9.0 but then decreases as the pH grows further. In general, a pH of 9.0 has been reported to produce the highest reaction rate, while a pH of 4.5 permanently inhibits urease activity. However, these values may vary depending upon the specific source and activity of the urease enzyme and might therefore require further investigation.

Cuccurullo et al. [81] demonstrated that the urease activity of a crude soybean extract rapidly reduces over time under room conditions, mainly because of a decrease in pH. After only a few hours of exposure to room atmosphere, the soybean extract stabilizes at a low pH value of 4.5, showing no signs of carbonate precipitation upon adding urea and calcium chloride. The storage of crude urease extracts can therefore be a major practical issue due to its rapid acidification, which may eventually impede enzymatic activity and, hence, the development of the precipitation reactions. To prevent this activity decay, the crude urease extract might be premixed with urea, whose hydrolysis would produce a stable nonacidic medium ready to precipitate carbonate upon addition of a calcium source.

Interestingly, the above results suggest that a cementing solution with a relatively low pH can delay the EICP reaction rate and, therefore, increase the spatial homogeneity of carbonate precipitation inside the treated ground, especially in large scale projects.

### 3.7. Effect of Soil Type

EICP research has predominantly focused on the treatment of coarse soils, with very few studies about fine soils. Oliveira et al. [107] applied EICP stabilization to five different soils, poorly graded sand with silt, two silty sands, sandy silt, and sandy silt with 11% organic content. Unconfined compression tests revealed an augmentation of strength after treatment of all soils except the organic sandy silt, which exhibited a decrease of strength accompanied by an increase of ductility. This decrease in strength and stiffness was attributed to the coating of soil particles with organic matter, which hindered the creation of intergranular carbonate bonds. The authors also speculated that the growth of carbonate crystals had broken some bonds inside the organic soil matrix, thus resulting in a deterioration of the mechanical properties after treatment.

Chandra and Ravi [108] performed unconfined compression tests on samples of silty sand, clayey sand, and silt previously stabilized via EICP. Results showed that the most significant strength gain was attained in the clayey sand, while the smallest gain was attained in the silt. The lesser enhancement of mechanical characteristics for the silt was attributed to a relatively acidic pH of 5. Oliveira et al. [107] observed instead significant strength gains in silt stabilized via EICP; however, it exhibited a larger pH of 7.75 as opposed to the lower pH of the silt tested by Chandra and Ravi [108].

Oliveira and Neves [109] mixed organic sand with a pH of 7.65 and an inorganic clay with a pH of 5.06 in different gradations to produce materials with variable organic content from 0% to 13%. The different materials were stabilized via EICP and subjected to unconfined compression tests, which showed larger mechanical gains at higher organic contents probably because of the greater pH values. The authors concluded that, with a pH value around 9, the effectiveness of EICP stabilization in coarse soils is not hindered by the presence of organic matter. Unfortunately, it is not possible to conclude whether an alkaline environment (pH around 9) has similar beneficial effects in organic silts and clays due to a lack of experimental evidence.

In summary, the pH of the reaction medium significantly influences the efficiency of the EICP reactions, even more than soil type and grading due to the small size of the urease molecule fitting inside pores with dimensions of tens of nanometers. This allows the application of EICP to the stabilization of silty and clayey materials [107,108,109], which is a fundamental advantage over MICP.

## 4. Mechanical Improvement of Soils by EICP Stabilization

### 4.1. Effect of EICP Stabilization on Strength and Stiffness

**Figure 12:**
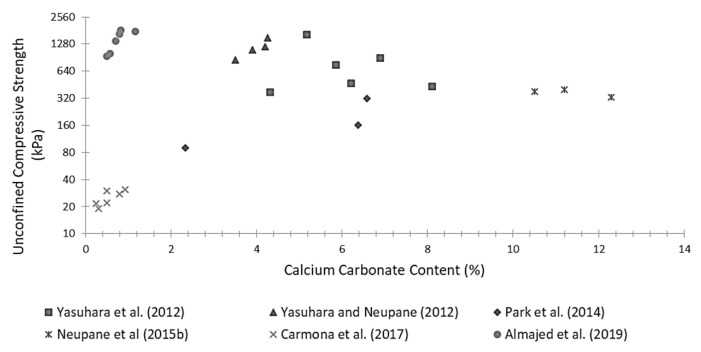
Data of Unconfined compressive strength versus calcium carbonate content from literature.

Numerous studies have reported significant improvements in the mechanical properties of soils stabilized via EICP [79,83,84,86,102,110]. Figure 12 shows published values of the unconfined strength measured on different types of treated soils containing variable levels of calcium carbonate (calculated as a percentage of the total soil mass). Figure 12 indicates that, in general, around 4% to 8% of carbonate content can consistently generate significant levels of strength in the range between 300 kPa and 2000 kPa. Interestingly, however, the largest strength of 1745 kPa, measured by Almajed et al. [110], at a low carbonate content of less than 1%, may be related to the growth of larger crystals at favourable nucleation sites provided by the presence of powdered milk inside the cementing solution. Conversely, Neupane et al. [102] reported an average strength of only 400 kPa at a much higher carbonate content, which is again explained by the sensitivity of the mechanical behaviour to the location of carbonate precipitation inside the material.

Most strength improvement is generated by the precipitation of carbonate at interparticle contacts, while the precipitation over the surface of the particles or within the pore space contributes minimally to the mechanical enhancement of the material. This aspect was experimentally investigated by Simatupang and Okamura [87], who conducted saturated undrained cyclic triaxial tests to measure the liquefaction resistance of EICP-treated sand samples cured at two different relative humidity levels of 30% and 97%. Scanning Electron Microscopy (SEM) analysis showed that the samples cured at the lower humidity exhibited crystallization of calcium carbonate at interparticle contacts as the little moisture inside the soil tended to accumulate at these locations. Conversely, the samples equalized at the higher humidity contained larger quantities of moisture flooding entire pores, which led to a different topology of carbonate precipitation coating the entire particles surface. Simatupang and Okamura [87] also observed that the samples cured at 30% humidity showed higher strength during cyclic loading than those cured at 97% humidity. Moreover, the liquefaction resistance largely depended on the strength of the interparticle carbonate bonds and, once these bonds were broken, it became very similar for both treated and untreated sand. Their experiments concluded that a volumetric strain of 1% significantly deteriorated carbonate bonds while a volumetric strain of 1.7% destroyed them.

**Figure 13:**
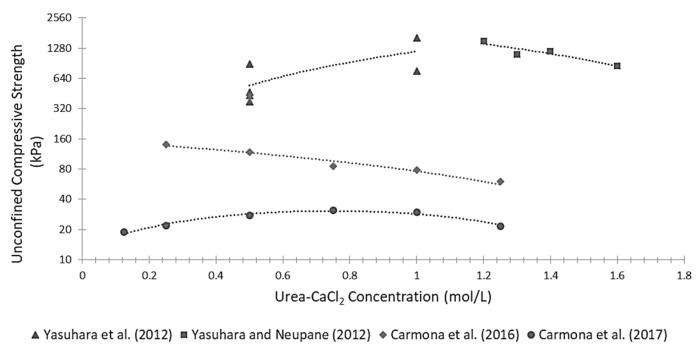
Data of Unconfined compressive strength versus urea–CaCl_2_ concentration from literature.

Figure 13 describes the variation of unconfined compressive strength with changing concentration of urea–CaCl_2_, recorded by different authors. Examination of Figure 13 shows that growth of the urea–CaCl_2_ concentration does not necessarily lead to a stronger material because an increase in urea brings a collateral pH reduction inside the cementing solution, which slows down the reaction rate. It might, therefore, be that the strength of the treated samples reduces as the reactants concentration increases beyond an optimum level, whose value depends on curing time and amount of urease.

Carbonate precipitation generally increases with growing urease content, though some authors [85,86] have observed a decrease in strength as urease concentration increases beyond a certain threshold. Li et al. [111] attributed this anomaly to the absorption of the urease enzyme onto the surface of the calcium carbonate precipitate, which limits crystal growth.

### 4.2. Effect of EICP Stabilization on Permeability

**Figure 14:**
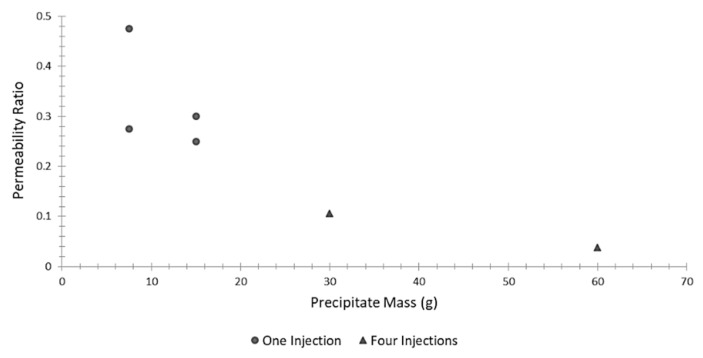
Permeability ratio of treated samples versus precipitate mass (Data from Yasuhara et al. [83]).

Only a handful of studies have quantified the reduction of soil permeability generated by EICP stabilization. Among these studies, Nemati and Voordouw [26] measured the permeability of water through stabilized sand columns, showing that an increase in urease concentration generates a corresponding reduction in the permeability ratio (i.e., the ratio of the final permeability, after treatment, to the initial permeability) from 0.9 to 0.6. The permeability ratio also decreased with an increase in curing temperature from 22 °C to 30 °C, though thermal effects become marginal at higher urease concentrations. Two- and three-fold increases in all reactants reduced the permeability by 23% and 31%, respectively, while three injection cycles of the cementing solution resulted in a decrease of permeability of 88%. Similar conclusions were reached by Yasuhara et al. [83], who conducted constant head permeability tests on EICP-treated sand samples. Four sand columns were treated with a single injection of the cementing solution, while two sand columns were treated with four injections performed at intervals of two hours. Figure 14 shows that permeability decreases with the increasing mass of precipitate and that the repeated injection pattern produces a significantly larger drop of material perviousness than a single injection. Hoang et al. [112] also measured the permeability of EICP-treated soil samples arriving at similar conclusions.

### 4.3. Effect of EICP Stabilization on Wind-Driven Surface Erosion

Wind-driven soil erosion poses a serious threat to human activities worldwide and is relevant to many technical fields, from geotechnical engineering to earth building. A prominent example is the mitigation of fugitive dust emissions from construction sites and mining areas, which may harm human health, especially in arid and semiarid environments. Fugitive dust emissions are usually controlled by applying clean water or aqueous salt solutions to the soil surface, though both these practices are unsustainable in the long term and might affect groundwater or vegetation. The application of synthetic polymers is a more effective and longer-lasting solution, albeit it remains relatively expensive. In this context, EICP treatment has shown great promise at a laboratory scale, but large scale studies are needed to confirm viability for industrial/commercial applications.

Hamdan and Kavazanjian [113] studied the surface erosion of EICP stabilized soil samples exposed to different wind velocities. The tests were performed inside a wind tunnel, and the erosion resistance was quantified via the measurement of the threshold detachment velocity, which is the wind velocity at which soil particles become entrained in the air stream. Three different soils were tested, well-graded fine sand (Sand Type 1), a uniform fine sand (Sand Type 2), and a mine tailing, which was also predominantly sand (Sand Type 3). The soil surface was sprayed with different cementing solutions containing a constant amount of urease equal to 0.45 g/L and variable concentrations of calcium chloride from 0.05 M to 2.0 M, while the ratio between urea and calcium chloride was kept constant at 1.5:1.

**Figure 15:**
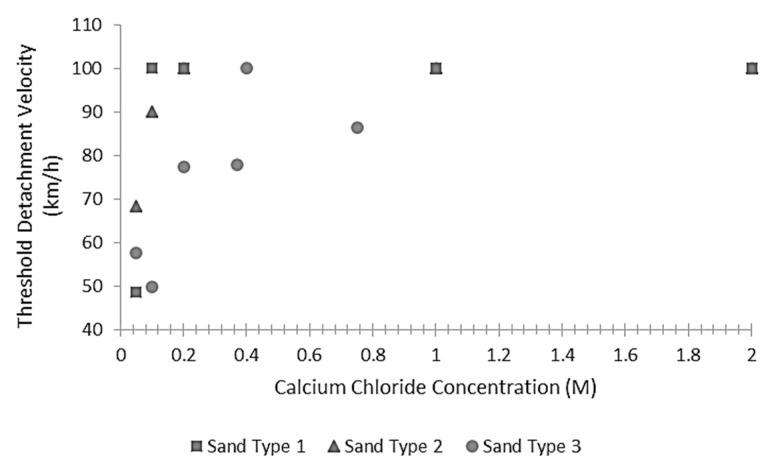
Threshold detachment velocity of treated samples versus reagent concentration (Data from Hamdan and Kavazanjian [113]).

The tests results revealed that the bare, dry soil had a threshold detachment velocity of 25.5 to 30.6 km/h, which increased to 82.8 km/h if the material was slightly moist. Figure 15 indicates that even a relatively small concentration of reactants produces a substantial increase of the threshold detachment velocity of the dry soil. Note that air velocities greater than 90 km/h could not be accurately measured due to limitations in the testing equipment and are therefore indicated as 100 km/h for illustration purposes. The well-graded and uniform fine sands (Sand Type 1 and Sand Type 2) consistently exhibited threshold detachment velocities higher than the measuring limit of 90 km/h, starting from minimal levels of reactants concentration, while the mine tailing (Sand Type 3) exhibited larger variance.

**Figure 16:**
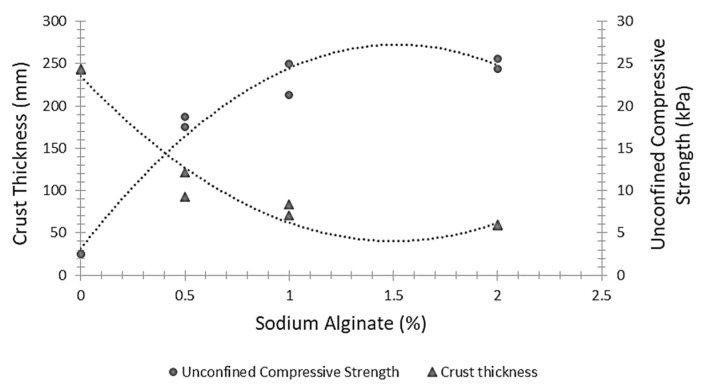
Approximate trends of crust thickness and unconfined compressive strength of treated samples against the percentage of sodium alginate (Data from Almajed et al. [116]).

The efficiency of the treatment is significantly reduced if the occurrence of EICP reactions on the exposed surface is substantially limited by the deeper material penetration or atmospheric evaporation of the cementing solution shortly after application. Researchers attempted to overcome the above shortcomings by increasing the viscosity of the cementing solution [114,115,116]. Hamdan et al. [114] added xanthan gum (powder), guar gum (powder) and polyol-cellulose (liquid) to the cementing solution while keeping the concentration of urea and calcium chloride constant at a ratio of 1.5:1. On average, the addition of polyol-cellulose generated a 2 mm thick crust on the surface of the treated samples, while both xanthan and guar gum generated a crust of about 10 mm thickness. The average penetration depth of the cementing solution was approximately 18 mm for xanthan gum, 15 mm for guar gum and 33 mm for polyol-cellulose. Vapour pressure tests revealed that the addition of xanthan gum generated the most significant increase in soil–liquid retention capacity while polyol-cellulose generated the smallest one. Almajed et al. [116] used sodium alginate, a natural polysaccharide derivative of alginic acid, to increase the viscosity of the cementing solution. They performed wind tunnel tests (under a maximum air velocity of 58.32 km/h) and hand penetrometer tests to measure the crust strength. All EICP stabilized samples showed a threshold detachment velocity higher than the measuring limit of 58.32 km/h, regardless of the amount of sodium alginate. It was also observed that the surface crust became thinner, more uniform, and stronger with an increase in the concentration of sodium alginate, as shown by the approximate trends of available data in Figure 16.

## 5. Numerical Simulation of EICP Stabilization

The numerical simulation of EICP and MICP reactions inside soils remains challenging due to the nature of these stabilization techniques. Modelling of these processes requires the integration of microbiological, chemical, hydraulic, and mechanical knowledge within a single analytical framework. Attempts at numerically replicating MICP processes have been made by some researchers [117,118,119,120], among others. Some attempts have also been made at simulating EICP processes to predict the changes in permeability and porosity in stabilized soils. Yasuhara et al. [83] used the non-isothermal reactive geochemical transport code TOUGHREACT [121] to model the advection–diffusion processes that occur during EICP stabilization. The results showed a good agreement between the computed and measured porosity variations during acid leaching tests, but the numerical predictions significantly overestimated the permeability measured during constant head tests. The code was unable to predict the buildup of calcium carbonate near the injection point and the accordingly low values of permeability measured during laboratory tests. The good agreement between the computed and measured values of porosity led, however, to the conclusion that the accuracy of predictions could be improved by introducing a more realistic dependency of permeability on porosity, grain size, tortuosity, and specific surface. Neupane et al. [85] reached a similar conclusion by comparing experimental data with numerical simulations from the same code. They highlighted that the reaction rate and reactants concentrations were significant factors affecting the predicted porosity changes.

Hommel et al. [122] developed a two-phase, multicomponent, non-isothermal reactive transport model using Darcy’s law to describe the porous flow. The model considered the effects of pH, temperature, fluid–fluid, and fluid–solid mass transfers, as well as the adsorption–desorption of urease and calcium carbonate. Comparison with experimental data indicated that the model was able to predict the concentration of the reactants only qualitatively and could not capture the spatially heterogeneous precipitation of calcium carbonate. These discrepancies were attributed to the uncertainties associated with geochemical parameters whose values were not found in the literature. A parametric study confirmed the soundness of the proposed model and emphasized the need for a comprehensive calibration under different experimental conditions.

The development of reliable computational models is important to supplement laboratory investigation and help predict field behaviour at a large scale. Future efforts must therefore be directed towards optimizing numerical models and refining parameter calibration to improve the accuracy of predictions.

## 6. Reduction of Toxic By-Products of EICP Stabilization

One of the downsides of the ureolytic stabilization of soils is the side production of ammonia and ammonium, which, in great quantities, may contaminate groundwater and increase greenhouse gas emissions. For example, Japanese law requires that industrial wastewater must not contain an ammonium concentration of more than 100 g/L [123]. Several methods have been proposed to remove NH-forms from soils (NH-forms is the collective term used for both ammonia and ammonium), including nitrification, ammonia stripping, chemical precipitation, and ion exchange [124,125].

**Figure 17:**
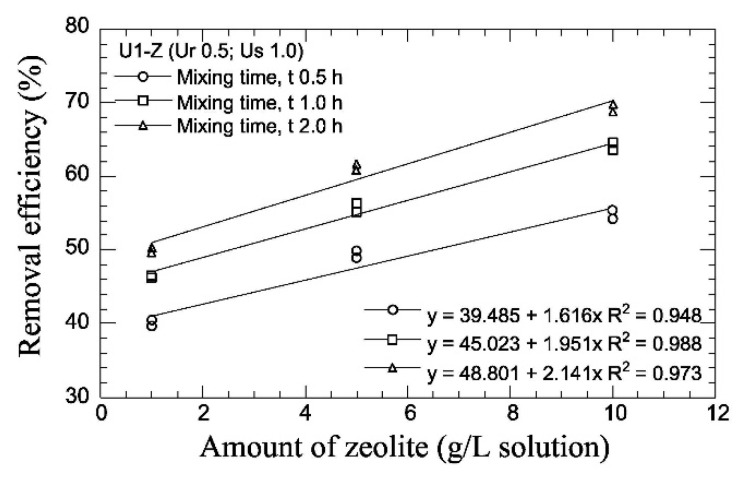
Efficiency of NH-forms removal by natural zeolite (from Putra et al. [126], Copyright under MDPI).

Zhao et al. [115] measured the ammonium removal ability of biomimetic cross-linked hydrogel via a colourimetric method using a UV spectrometer. They found a maximum ammonium removal of 96%, though their experiments did not involve any soil, and no conclusion can, therefore, be made on the removal efficiency in real conditions. Putra et al. [126] attempted to reduce the NH-forms resulting from EICP by mixing a urease-urea solution with variable amounts of natural zeolite, which exhibits high cation exchange capacity, cation selectivity, higher void volume and a great affinity for ammonium [127,128,129,130]. The solution was then filtered to remove the zeolite and was mixed with a pH adjusting agent containing sodium hydroxide to convert all NH-forms into ammonia, which was then measured by selective electrodes. Zeolite concentration and mixing time were found to significantly affect the decrease in NH-forms, attaining a maximum removal efficiency of almost 70% (Figure 17). The addition of calcium chloride after the ammonia measurement also showed higher levels of carbonate precipitation at larger zeolite contents.

## 7. Conclusions

The past two decades have seen a tremendous increase in research about earthen construction and sustainable soil improvement techniques, both of which can curb the exploitation of natural resources and the emission of greenhouse gases. In this context, enzymatic-induced carbonate precipitation (EICP) has attracted the attention of geotechnical engineers and geomicrobiologists as an environmentally friendly and viable soil stabilization method with multiple engineering applications. This paper presents a state-of-the-art review of some of the major research advances in EICP stabilization, focusing on the parameters that affect reaction rate, chemomechanical processes, the scale of ground improvement, and mitigation of toxic by-products. Some of the key takeaway points of this article are listed below.

- EICP treatment on a large scale may lack spatial homogeneity due to a quick reaction rate, causing the formation of fresh precipitate near the injection point and restricting the transport of the cementing solution to relatively long distances. This can be circumvented by controlling the reaction rate, which is closely related to reactants concentration, temperature, and pH of the cementing solution. However, understanding the effects of these factors is required to efficiently control the EICP reaction rate.
- The strength of EICP-treated soils largely depends on the distribution of nucleation sites inside the granular assembly, with carbonate precipitation at interparticle contacts being most effective in improving the mechanical characteristics of the material, whereas carbonate precipitation on the surfaces of the particles has negligible effect.
- The activity of urease determines its amount to be added to the cementing solution relative to other reactants in order to achieve a specific degree of precipitation in a target curing period. Ureases from different sources have variable degrees of activity which, under fixed levels of pH, temperature, and reactant concentrations, lead to the precipitation of different amounts of calcium carbonate at different reaction rates.
- For given levels of enzymatic activity and reactants concentration, a larger amount of urease produces greater carbonate precipitation and increases reaction rate for a fixed curing period. For a constant urease activity and curing period, a proportional increase of all reactants’ concentrations increases calcium carbonate precipitation, with a marginal increment of reaction rate.
- Increasing reactants concentrations beyond a certain level, with constant urease amount and curing period, however, reduces reaction rate and amount of precipitate, most likely due to the drop of pH provoked by the increase of urea. If the curing period is extended, a precipitation ratio of 100% may be attained even for high reactants concentration relative to the amount of urease.
- A rise in temperature, up to a certain level, increases urease activity and, hence, reaction rate, with the maximum degree of activity being observed at different temperatures depending on the specific source of the enzyme. Likewise, the very high or extremely low pH of the cementing solution decreases the reaction rate; the optimum pH level has been reported at around 9.0.
- Crude plant extracts provide a viable and economical alternative to chemically purified urease, but their enzymatic activity rapidly declines over time, making their stable storage an important area of investigation.
- Considerable improvements in material strength have been reported for treated soils containing a relatively small amount of calcium carbonate, from 4% to 8%. The introduction of organic reactants, such as nonfat milk, can promote larger crystal growth due to the introduction of nucleation sites and demonstrate very high strength, up to 1.75 MPa, with a small amount of calcium carbonate below 1%.
- A volumetric strain of about 1% can produce significant damages to interparticle cementation, while a volumetric strain of 1.7% causes the complete destruction of the carbonate bonds. After bonds breakage, the difference of mechanical behaviour between treated and untreated soils becomes negligible.
- The physicochemical transformation of the soil during EICP treatment has been undertaken using advection–diffusion computer codes resulting in relatively accurate predictions of porosity changes while permeability values were largely overestimated.
- The introduction of additional reactants, such as magnesium chloride and magnesium sulfate, alters the precipitation rate and transforms the mineralogy of precipitate from crystalline to amorphous.
- Experimental evidence indicates that EICP can also improve the mechanical characteristics of both inorganic finer soils and organic coarser soils, provided that the pH of the reaction medium is not acidic. EICP is reported to be impractical in organic soils of low pH as a low pH inhibits urease activity and the biological coating of the soil particles hinders carbonate bonding.
- Laboratory experiments have shown that introducing natural zeolite inside the cementing solution can reduce toxic by-products of EICP reactions such as NH-forms by a relatively large margin up to 70%.

It has also emerged that some critical aspects of EICP stabilization have not been sufficiently explored, such as, for example, the sensitivity of carbonate precipitation to acidic environments and the effect of organic content on the efficiency of the treatment, especially in fine-grained materials. Equally, the response of treated soils to hydraulic, hygroscopic, and environmental changes, including the response of the stabilized earth to wetting-drying or freeze-thaw cycles, is an overlooked area of research that demands greater attention.

## 8. Future Research Foci for the Enzymatic Stabilization of Soils

It is anticipated that future research on EICP should focus on different aspects to improve this method’s applicability in mainstream geotechnics, as listed below.

- The effect of the pH of the reaction medium on the treatment efficiency should be investigated to assess the viability of soil stabilization in different environmental contexts. For example, the solubility of the carbonate precipitate in acidic media must be understood if the technique is to be used in practice.
- A database of the necessary curing times to attain a precipitation ratio of 100% for different reactants concentrations and urease activity levels should be established to relieve geotechnical engineers from carrying out preliminary precipitation tests at the start of each campaign.
- In addition to studying the soil enhancement in terms of strength, stiffness, and permeability, the future investigation should concentrate on the spatial homogeneity of the improvement, especially in large scale projects. Research efforts should therefore be directed towards the optimization of the pH, temperature, and concentrations of the reactants in cementing solution to attain slower reaction rates that can delay carbonate precipitation and consequently allow the treatment of larger areas.
- Accurate numerical and constitutive models should be developed to better understand and predict the physicochemical changes undergone by the soil during stabilization, with a focus on the changes of porosity, permeability, strength, and stiffness.
- Small scale laboratory studies have demonstrated the potential efficacy of surface treatment for controlling fugitive dust emissions and the erosion of earthworks. Nevertheless, large scale tests must be performed to validate the durability of surface stabilization before application to real problems.
- The environmental impact of potentially toxic by-products of the stabilization reactions, e.g., NH-forms, must be quantified, and, if necessary, viable mitigation measures must be proposed.
- Research efforts must be directed towards assessing the resistance of the treated ground to thermal, hydraulic, hygroscopic, and mechanical actions, which is necessary to understand the viability of this stabilization technique in different climatic conditions.
- Future EICP research must also explore new methods of urease extraction (chemical or physical) and optimize the currently known ones to decrease the bottlenecks associated with urease mass production (mainly cost and long-term storage) if EICP is to be used in large-scale construction and mining projects.

## Figures and Tables

**Table 1:** List of main publications reviewed.

Articles	Urease Source (Activity)	Parameters Affecting EICP Reaction							Effect of EICP on Soil Properties			Supporting Research	
		Reactants Concentrations	Temperature	pH	Magnesium Salts	Urease Source	Calcium Source	Soil Type	Strength and Stiffness	Permeability	Surface Treatment	Numerical Modeling	Toxic by-Products
Nemati and Voordouw (2003)	Jack beans (2610 U/g)	**X**	**X**							**X**			
Yasuhara et al. (2012)	Jack beans (N/A)	**X**							**X**	**X**		**X**	
Yasuhara and Neupane (2012)	Jack beans (2950 U/g)								**X**				
Hamdan et al. (2013)	N/A								**X**				
Neupane et al. (2013b)	Jackbeans (2950 U/g)	**X**							**X**	**X**		**X**	
Nam et al. (2014)	Jackbeans extract					**X**							
Park et al. (2014)	Jackbeans extract						**X**						
Neupane et al. (2015b)	N/A		**X**										
Kavazanjian and Hamdan (2015)	Jackbeans (200 U/g)								**X**				
Zhao et al. (2016)	Jackbeans (15,000–50,000 U/g										**X**		**X**
Carmona et al. (2016)	Jackbeans (34,310 U/g)								**X**				
Putra et al. (2016)	Jackbeans (2950 U/g)				**X**								
Oliveira et al. (2016)	Jackbeans (34,310 U/g)							**X**					
Hamdan and Kavazanjian (2016)	Jack beans (2610 U/g)										**X**		
Hamdan et al. (2016)	Jack beans (2610 U/g)										**X**		
Dilrukshi and Kawasaki (2016)	N/A												
Putra et al. (2017a)	Jackbeans (2950 U/g)				**X**								
Putra et al. (2017b)	Jack beans (2950 U/g)												**X**
Simatupang and Okamura (2017)	N/A	**X**							**X**				
Carmona et al. (2017)	Jackbeans (34,310 U/g)	**X**							**X**				
Hoang et al. 2018	S. pasteurii sonification					**X**				**X**			
Almajed et al. (2018)	N/A (3500 U/g)								**X**				
Chandra and Ravi (2018)	Jack beans (40,150 U/g)							**X**					
Dilrukshi et al. (2018)	Watermelon Seeds		**X**			**X**							
Almajed et al. (2019)	N/A (3500 U/g)								**X**				
Cuccurullo et al. (2019a)	Soybean extract					**X**							
Cuccurullo et al. (2019b)	Soybean extract					**X**							
Rohy et al. (2019)	Jackbeans (1500 U/g)			**X**									
Oliveira and Neves (2020)	Jackbeans (34,310 U/g)			**X**				**X**					
Hommel et al. (2020)	N/A											**X**	
Cui et al. (2020)	N/A			**X**									
Almajed et al. (2020)	Jackbeans (1500 U/g)										**X**		
Sun et al. (2020)	Jackbeans (1030 U/g)		**X**	**X**								**X**	
Baiq et al. (2020)	Cabbage and Soy pulp extract					**X**							

## Data Availability

No new data were created or analyzed in this study. Data sharing is not applicable to this article.
